# Notes and News

**Published:** 1904-07-23

**Authors:** 


### NOTES AND NEWS.

The Duchess of Saxe-Coburg and Gotha, patroness, visited the Hospital for Epilepsy and Paralysis in Maida Vale last week. Princess Henry of Battenberg, who accompanied her, consented to become a patroness of the hospital.

President Loubet has unveiled the monument erected to Pasteur's memory in the Place Breteuil, Paris.

An endeavour is being made to raise £35,000 for the purpose of establishing and endowing a Home of Recovery, with 21 free beds, and four for paying patients. Mr. R. B. Martin is the treasurer.

A Russian hospital train to run between Irkutsk and Kazan has been despatched from Kieff. The hospital trains are arranged on a uniform system. The staff of each consists of three or more fully qualified doctors, from six to ten Red Cross sisters, and from 30 to 40 hospital assistants. The accommodation is from 200 to 300 patients.

Mr. John Tweedy, F.R.C.S.Eng., ophthalmic surgeon to University College Hospital, has been re-elected President of the College of Surgeons, and Mr. A. Mayo Robson, F.R.C.S.Eng., Emeritus Professor of Surgery, Yorkshire College, and consulting surgeon, Leeds General Infirmary, and Mr. Henry T. Butlin, F.R.C.S.Eng. and D.C.L. Durham, consulting surgeon to St. Bartholomew's Hospital, have been elected vice-presidents for the ensuing year.

The League of Mercy has been very active recently, and a most successful drawing-room meeting was held at Bishop's Hall, Romford, by the kind invitation of Colonel Lockwood, M.P., and Mrs. Lockwood, President and Lady President of the Epping District. Lord and Lady Farquhar, President and Lady President of the West Marylebone District, held a most successful meeting of the Marylebone Districts on Monday, July 11th, at 7 Grosvenor Square, W. By kind permission of the Archdeacon of Rochester and Mrs. Cheetham, President and Lady President of the Rochester District, a well-attended drawing-room meeting was held at "The Precincts," Rochester, on July 14th.

The cholera mortality in Persia shows some decrease, but the number of deaths is still very large.

The new buildings of the North-Eastern Hospital for Children were opened by the Princess Louise last week. The new premises consist of four wards of 11 beds, one for six, and one for seven; a new operating theatre, and a casualty room. The cost of the enlargement has been £45,000, of which £9,000 requires to be met.

The Ophthalmological Society of the United Kingdom have issued a protest against the expressed intention of the Company of Spectacle Makers to issue a certificate for sight testing. The society points out that such a course might prove dangerous to the public, as defective eyes, any more than any other defective organ of the body cannot be safely treated by those unacquainted with anatomy, physiology, and various morbid conditions.

A conference has been held between the Medical Defence Union, the Medico-political Committee, the London and Counties Medical Protection Society, and the South-west London Medical Society on the subject of the Relations between Coroners and the Medical Profession. The proceedings adopted by Mr. Troutbeck form the text of the objections raised. It being reported that Mr. Troutbeck gives medical men scant opportunity to attend the inquest for their patients, and failing their attendance owing to short notice he makes reflections to their discredit. It is further stated that he entrusts a large number of post-mortem examinations to a pathologist who is not generally recognised as an expert. Complaints were made more than a year ago, but as no steps appear to have been taken the conference intends to ask for the interference of the Prime Minister.

A very long list of honorary consulting medical officers to King Edward VII.'s Hospital for Officers has been published. It includes the following names:—Sir Thomas Smith, Bart., K.C.V.O., F.R.C.S.; Sir Frederick Treves, Bart., K.C.V.O., C.B., F.R.C.S., LL.D.; Dr. Charles A. Morris, C.V.O., M.A., M.C., F.R.C.S.; Mr. Herbert W. Allingham, F.R.C.S.; Sir Thomas Barlow, Bart., K.C.V.O., M.D., F.R.C.P., LL.D., D.Sc.; Sir William Henry Bennett, K.C.V.O., F.R.C.S.; Mr. Tom Bird, M.A., M.R.C.S.; Mr. Anthony A. Bowlby, C.M.G., F.R.C.S.; Sir William H. Broadbent, Bart., K.C.V.O., M.D., F.R.C.P., F.R.S., LL.D.; Mr. Arthur H. Cheatle, F.R.C.S.; Mr. G. Lenthal Cheatle, C.B., F.R.C.S.; Sir Anderson Critchett, F.R.C.S.Edin.; Dr. Alexander Crombie, C.B.; Dr. David Ferrier, F.R.C.P., F.R.S., LL.D.; Mr. P. Johnston Freyer, M.A., M.D., M.Ch.; Sir Alfred Downing Fripp, C.V.O., C.B., M.S., F.R.C.S.; Mr. Rickman Godlee, M.S., F.R.C.S.; Dr. James Frederick Goodhart, F.R.C.P., LL.D.; Dr. Frederick William Hewitt, M.V.O., M.A.; Sir Victor A. H. Horsley, B.S., F.R.C.S., F.R.S.; Mr. George Henry Makins, C.B., F.R.C.S.; Mr. Percy Lockhart Mummery, M.B., B.C., F.R.C.S.; Dr. Frederick W. Pavy, F.R.C.P., F.R.S., LL.D.; Sir Richard D. Powell, Bart., K.C.V.O., F.R.C.P., M.D.; Dr. William Aldren Turner, F.R.C.P.; Mr. Henry Roe Walker, M.R.C.S., L.R.C.P.; Dr. William Hale White, F.R.C.P.

